# Implementation of the Nagoya Protocol within the Collection of Institut Pasteur

**DOI:** 10.1099/acmi.0.000008

**Published:** 2019-04-16

**Authors:** Raquel Hurtado-Ortiz, Alexandra Hébreu, Evelyne Bégaud, Chantal Bizet-Pinson

**Affiliations:** 1 CRBIP – Biological Resource Centre, Institut Pasteur, 25-28 rue du Dr Roux 75015, Paris, France; 2 CNCM – Collection Nationale de Cultures de Microorganismes, Institut Pasteur, 25-28 rue du Dr Roux 75015, Paris, France; 3 BioSpeedia, Institut Pasteur, 25-28 rue du Dr Roux 75015, Paris, France; 4 CIP – Collection of Institut Pasteur, Institut Pasteur, 25-28 rue du Dr Roux 75015, Paris, France

**Keywords:** Nagoya Protocol, microbial biological resource centres, microbiology

## Abstract

The focus of the EU regulations on the Nagoya Protocol on Access and Benefit-Sharing leaves the control of access to genetic resources up to each member state. France has chosen to control access and is going to put in place regulations for it. All the materials received should have specific documentation regarding the accession of genetic resources, where there is a National Authority to issue them. The European commission will maintain a list of biological collections with registered status proposed by each country. The member states are responsible for considering inclusion and verification of these collections. In recent years, the Collection of Institut Pasteur (CIP) staff has expressed concern over how to interact with the implementation of the Nagoya Protocol in the collection but also at the national level with the aim that the CIP will be a registered collection. The advantage of accessing resources from a registered collection is that users of genetic resources will be considered as having exercised ‘due diligence’ if they source their genetic resources from these collections. This could facilitate the process for scientists when applying for research funding. The CIP organized the accession of new deposits and the distribution of micro-organisms in connection with it.

## Introduction

Regulations on Access to Genetic Resources and the fair and Equitable Sharing of Benefits Arising from the Utilization to the Convention on Biological Diversity (CBD) leaves the control of access to genetic resources up to each member state to establish their own national legislation with the goal of ensuring that benefits of genetic resource utilization are shared equitably with provider nations. The fair and equitable sharing of the advantages stemming from the use of the genetic resources and the associated traditional knowledge, aim to restore some equity between suppliers and users of these resources and knowledge and contributes to the preservation and the long-sustainable use of the biodiversity. Every state has sovereign rights over their natural resources and can be a supplier and user. It determines rules to obtain and use the genetic resources under sovereignty and takes measures assuring the fair and equitable benefit-sharing stemming from the use of the resources.

The Nagoya Protocol (NP) on Access and Benefit-Sharing (ABS), an international agreement adopted in 2010, which came into force on 12 October 2014, was the result of several years of negotiations that started with some countries which claimed biopiracy (Biopiracy happens when researchers or research organizations take biological resources without official sanction, largely from less affluent countries or marginalized people). The most important goal of the implementation of the Nagoya Protocol is to build confidence and relationships between the North and the South countries, particularly in research and development.

The European Union legislation, EU regulation no. 511/2014 on ABS, emphasizes that it is applicable to genetic resources from countries that have ratified the Nagoya Protocol, exercise sovereign rights, and have established ABS measures. All the genetic resources collected in EU members countries and used in research and development should comply with these measures. However, states can also decide whether or not to leave their resources in free access. Countries such as France and Spain are implementing regulatory control of access.

At the moment of writing, 110 states worldwide have ratified the Nagoya Protocol and various legal frameworks for implementing it may exist according to countries. The information regarding national requirements and procedures for access to genetic resources and associated traditional knowledge (TK) is managed in a central depository, the Access and Benefit-Sharing Clearing-House (ABS-CH) (https://absch.cbd.int/) [1]. The information includes legislative, administrative or policy measures, national focal points and competent authorities responsible for providing information on accession to genetic resources and documents associated to them, such as prior informed consent (PIC), mutually agreed terms (MAT) and the Internationally Recognized Certificate of Compliance (IRCC), proving that the access requirements of the provider country have been properly met.

### Microbial Biological Resource Centres, a key actor for compliance with NP in microbiological research

The work carried out at the global level by the Organization for Economic Cooperation and Development (OECD) has resulted in a definition of the Biological Resource Centres (BRCs) as well as the setting up of recommendations for their structuring and management. BRCs are specialized centres that collect, validate, study, secure and distribute collections of living organisms (seeds, grafts, micro-organisms, etc.) and ‘replicable’ parts of these organisms (DNA banks, plasmids) under rigorous conditions of quality and traceability; they also maintain the databases associated with these collections. BRCs are essential elements of the international research system, especially in the field of biotechnology. They also contribute to the preservation of biodiversity and genetic resources in the context of national policies and international conventions [2].

Microbial Biological Resource Centres (mBRCs) are dedicated to the preservation and enhancement of microbial diversity. They group micro-organisms either generally, with a maximum representation of biodiversity, or in a more specialized way, either in the form of thematic selections, yeasts, filamentous fungi, food bacteria, human or animal pathogenic bacteria, bacteria associated with plants, entomopathogenic bacteria, etc.

The main missions of mBRCs are:

- to ensure the sustainability of collections of micro-organisms by means of their perfect characterization, maintenance and preservation;
- to enrich its collections by exploring the biodiversity of micro-organisms of interest by collecting various biotypes from original strains;
- to scientifically and economically exploit these collections by ensuring their widest possible dissemination to the academic, scientific and industrial community by associating them with services (identification, characterization, and secured deposit, among others).

The Institut Pasteur hosts more specialized micro-organism collections (bacteria, fungi, viruses, parasites) on a single site than any other institution in France. Most of these collections are housed in and maintained by research laboratories. Only a few of these collections are ‘open’, distributing strains to analytical, industrial and research laboratories, other laboratories and teaching establishments on request. The largest of these collections, the Collection of Institut Pasteur (CIP) contains more than 15 000 bacterial strains and almost 130 plasmids. In December 2001, the Institut Pasteur undertook a project to create a BRC, the CRBIP (Centre de Ressources Biologiques de l’Institut Pasteur) as defined by the OECD, incorporating its collections of micro-organisms.

The aim of the CRBIP was to harmonize the management of collections on the campus, to enrich collections in an organized manner (e.g. with strains from collections of research laboratories), to ensure that the strains or products stored are characterized as thoroughly as possible, to improve conservation conditions (in two forms, at two different locations) and to ensure the distribution of samples according to health and environmental safety norms, in accordance with the laws and regulations in force. Quality assurance has been an integral part of this project since its inception, to meet the criteria laid down by the OECD [2]. The quality assurance manual produced describes the quality system established for the CRBIP, focusing on the process approach for activities. Six processes are described: integration of a collection into the CRBIP, acquisition, conservation and distribution of biological material, mastering the quality management system and bringing the strategy to life. mBRCs facilitate access to validated micro-organisms to promote reproducibility in science and provide raw material for new areas of inquiry [3].

Most of these collections hold materials that were isolated within the sovereign boundaries of another country. In order to enable due diligence, the European Commission have proposed a list of registered collections in order to guarantee that users who source their genetic resources from these registered collections, will be considered as having exercised ‘due diligence’.

In recent years, the CIP staff has expressed concern over how to deal with the implementation of the EU regulation of the Nagoya Protocol but also with the French regulatory control of access, which is not well-defined yet.

### Implementation of the Nagoya Protocol within the Collection of the Institut Pasteur

The adoption of the Nagoya Protocol in October 2010 has an obvious impact for mBRCs and, therefore, for the Collection of Institut Pasteur (CIP). Since the ratification by France in August 2016, the application of the NP is binding on all users of genetic resources established on the national territory. The CIP, as mBRC, is considered to be a user of genetic resources, and for this reason, the first steps to comply with the Nagoya Protocol have been implemented.

During the first stage of the implementation of the European regulation at the CIP, different tasks have been studied and others have been carried out:

- inventory of genetic resources linked to the NP;
- verification of information and documents related to genetic resources;
- exchanges of emails with depositors of microbial strains: requests for important information and documents on genetic resources, where necessary;
- drafting an explanatory sheet on general information regarding the Nagoya Protocol;
- archiving of information collected on genetic resources and storage for at least 20 years;
- verification of the procedure applicable to the CIP;
- verification of compliance of newly deposited genetic resources.

At the European level, collections and mBRCs are obliged to respect the due diligence. Therefore, in order to establish its compliance with international provisions, the CIP must undertake the traceability of these genetic resources and then be registered in the EU Register of Collections. Generally speaking, traceability involves gathering all the essential information and documents relating to genetic resources.

As part of this compliance, an inventory of all genetic resources (which are mainly bacterial strains) deposited within the CIP, has been carried out since 12 October 2014 (date the Nagoya Protocol came into force). Due to the large number of new strains received in the CIP each year, the first part of the inventory was performed with the genetic resources received between 12 October 2014 until 30 December 2015. From January 2016, the deposit form was modified with the aim of including information regarding ABS issues and compliance with the NP.

The following specific information regarding the biological material was studied and if necessary, the CIP asked the depositors to complete the information regarding the Nagoya Protocol:

- the date of deposit in the CIP;
- the name of the depositor and his/her host institution;
- the depositor identification number of the strains as well as the CIP identification number;
- the date of *in situ* isolation and/or *ex situ* isolation (where applicable);
- the place of isolation (including country of origin) of original collecting *in situ*;
- the name of the person who isolated the strain and his/her host institution

In total, 36% of the strains received in the period before mentioned, had incomplete information, such as the date of isolation, the place of *in situ* isolation or the name of the person (or institution) who isolated the strain. Thus, these resources were not considered to be in compliance with Article 4 of the 511/2014 Regulation. With the aim of respecting the due diligence, it was decided to conduct a retroactive compliance contacting the depositors of these genetic resources by e-mail in order to request the specific missing information, as well as documents such as PIC, MAT and/or the IRCC. Nevertheless, since most scientists were not aware of the Nagoya Protocol, an explanatory note on the international instrument was also included in the e-mail in order to inform them about the legal context of the NP and to explain the reasons why the CIP requested such information. At the time of writing this article, several depositors replied sending the information requested for 77% of the 36% of the strains with incomplete information. The depositors of the other 23% strains have not replied yet, despite having been contacted several times. The written reply of depositors was printed and scanned to be kept as proof of due diligence. However, none of the depositors contacted had PIC, MAT and/or the IRCC documents.

**Fig. 1.**
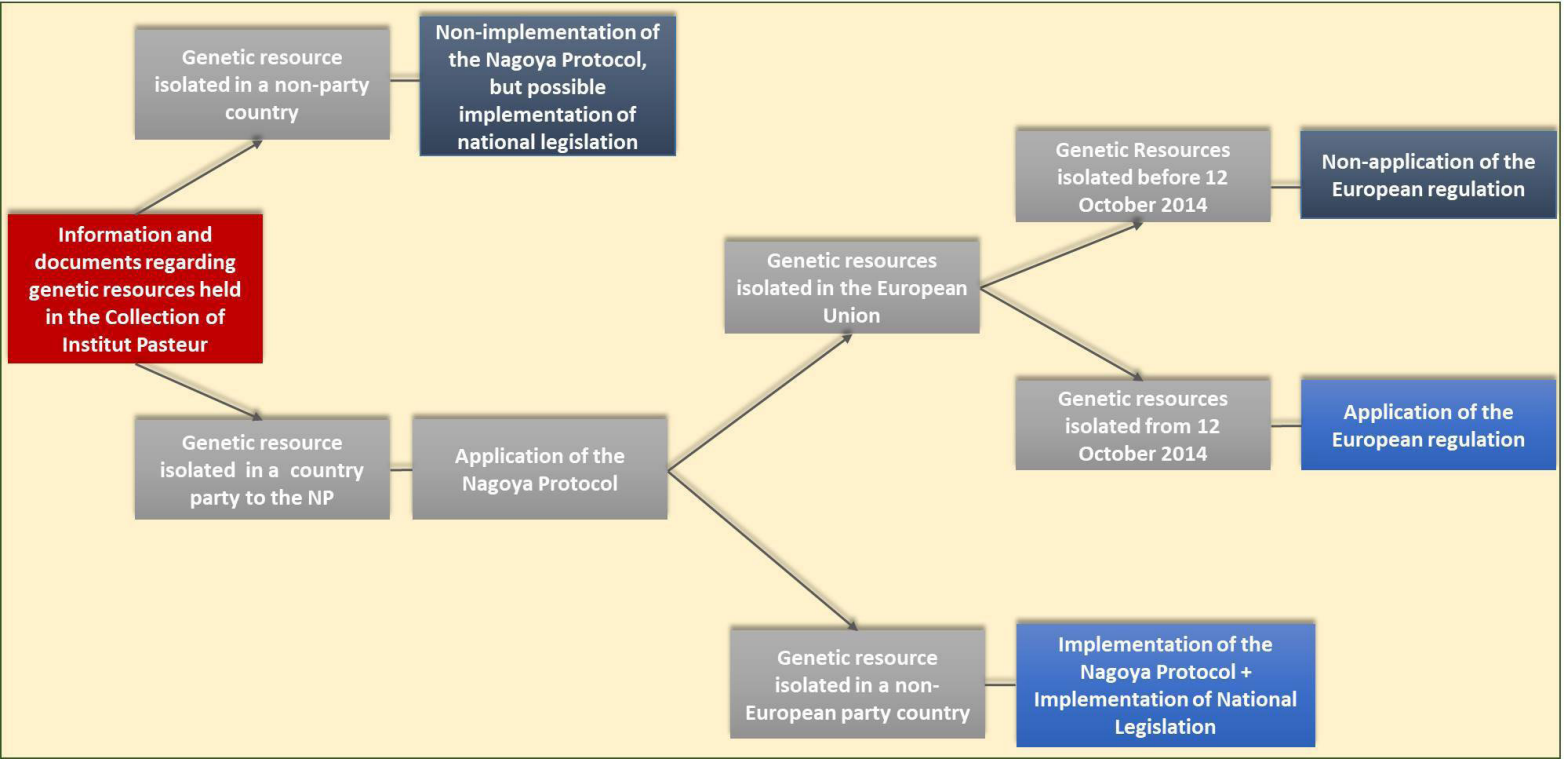
Scheme of the implementation of the European regulation of the Nagoya Protocol in the Collection of the Institut Pasteur.

According to our experiences during the implementation of the Nagoya Protocol in the CIP, the procedure to be followed to verify the compliance of a deposited genetic resource is explained in Fig. 1.

### Bottlenecks and practical cases when depositing strains in the CIP

Deposition of microbial resources in public mBRCs facilitates their availability, the confirmation of findings and allows continuity of research projects. Strategies have been devised to encourage researchers to deposit a higher fraction of the strains they work with and, at the same time, to implement quality measures and to comply with regulations. However, the necessary expansion of human resources and infrastructure is moving slowly.

Based on a daily experience on receiving strains coming from different geographical regions, we describe here four real facts experienced during recent deposition of bacterial strains in the CIP:

1. Deposit of strains with the necessary information, but without ABS associated documents. This may be considered as a partial compliance with the NP.
2. At the time of writing the article, only a few countries were able to issue an IRCC (Belarus, Bulgaria, Dominican Republic, Guatemala, India, Kenya, Malta, Mexico, Panama, Peru, South Africa and Spain). Thus, the CIP cannot request this document if one of its depositors does not have any document relating to a genetic resource originating from a country other than the countries above mentioned.
3. Genetic resources isolated from country parties to the Nagoya Protocol. When the information and documentation, regarding genetic resources, are not completed, depositors are contacted to obtain such information and, in this way, restore compliance. However, there are cases when depositors do not reply to the email or are unable to give us the information or the associated documents, since even they do not have this information.
4. Genetic resources from country parties of the NP may have their own legislative, administrative or policy measures. This is the case of two genetic resources isolated in Spain, which adopted the regulation 124/2017 of 24 February 2017 on access to genetic resources of wild taxa and control of its use. The Spanish regulation states that when the resource is used or distributed only for taxonomic purposes, a permit is not required. This regulation applies to biological resources isolated in Spain. Thus, in this case, depositors are not obliged to issue a PIC, MAT and/or an IRCC since the CIP has only performed taxonomy with them. However, the biological material within the CIP is not only redistributed for taxonomic purposes, but for research or industrial applications, for example. Therefore, according to the Spanish regulation, a permit must be obtained by the CIP directly with the competent Spanish authority. In order to establish compliance of the genetic resources, the Directorate General of Quality and Environmental Assessment and Natural Environment (Spanish Competent Authority) was contacted by email. However, there are difficulties in accessing information on regulations of a third country, such as when a document is written in a foreign language, without translation into English.

The EU Regulation 511/2014 imposes several obligations on users of genetic resources or traditional knowledge. According to the article 4 Reg. 511/2014 users are obliged to exercise due diligence to ascertain that: (a) genetic resources and traditional knowledge have been accessed in accordance with applicable access and benefit-sharing legislation or regulatory requirements, and (b) benefits are fairly and equitably shared upon mutually agreed terms, in accordance with any applicable legislation or regulatory requirements. For these purposes, users have to seek, keep and transfer to subsequent users the PIC, MAT and/or the IRCC.

Our experience in such a time-short exercise highlighted the difficulties in obtaining information related to the due diligence associated with access to genetic resources. Even more, there are specific national legislation in different countries which state other meanings for terms such as the term ‘access’. For example, Brazil has taken the position that access to all genetic resources from Brazil does not begin when they were collected, exported and deposited in collections, but when research and development activities (access) take place [4]. On the other hand, some countries like the USA have not ratified the Nagoya Protocol. Nevertheless, many collections in the USA hold materials that were isolated within the sovereign boundaries of another country. If this accession was made prior to the ratification of the CBD or the Nagoya Protocol, there is no guarantee that the USA should not comply with the NP, if retroactivity is approved for some national legislation, for example, the French national legislation [5].

Other concerns are focused on the possibility of involving digital sequence information in the decisions of parties of the NP. During the Second Meeting of the Parties to the NP (COP/MOP 2), held in December 2016, some issues remained undefined and a comprise proposal was adopted by COP/MOP 2 to establish a 2-year process to consider the potential implications of digital sequence information for the three objectives of the CBD. The outcome of this process was considered during the third Meeting of the Parties at the end of 2018.

Benefit-sharing of biological resources typically focus on the valuation of the presence of a particular micro-organism on state territory and the resulting ownership from the micro-organism *in situ* by the state [6]. In this sense, we have to emphasize that mBRCS and collections do not claim any downstream ownership rights on the microbial materials they keep in custody as service for the academia, research and industry. However, the implementation of the NP requires a clear tracking and monitoring of the access agreements and of the utilization of the accessed material by all the parties involved in international exchanges, which dramatically increase the transaction and operation costs [7].

With the purpose of dealing with the difficulties and bottlenecks that the NP brings to collections, BRCs, research laboratories, R and D institutions, etc., some best practice documents have been delivered by European Consortia such as CETAF (Consortium of European Taxonomic Facilities). Focusing on mBRCs and microbial collections, as well as, research laboratories on microbiology, some have already implemented a system in order to comply with the tracking and monitoring obligations of the NP, such as the European Culture Collections’ Organisation standard MTA (www.eccosite.org). Also, the Best Practice Manual on Access and Benefit Sharing of the Microbial Resource Research Infrastructure (MIRRI), provides guidance for implementing ABS policies and working procedures for acquisition of microbial material, accession in public collections, transfer of material to third parties and the delivery of other services (www.mirri.org) [7, 8]. Therefore, the investment in codes of conduct and guidelines that integrate ABS concerns seems a crucial component for a mutually supportive implementation [7].

### Conclusion

The application of the Nagoya Protocol in the mBRCs and microbial collections faces several difficulties: (a) the implementing regulations can be very specific in each country; (b) the complexity in collection of retroactive data and information regarding the origin of the genetic resources could be overwhelming; (c) difficulties linked to the time in creating and writing the necessary documents in signatory countries could harm goodwill in complying with the established regulations; (d) bureaucracy in obtaining such documents could hamper accession and provision of mBRCs and collections, which in turn could hamper scientific work using microbial genetic resources.
